# Tumor Associated Macrophages, as the Dominant Immune Cells, Are an Indispensable Target for Immunologically Cold Tumor—Glioma Therapy?

**DOI:** 10.3389/fcell.2021.706286

**Published:** 2021-07-21

**Authors:** Ni Tong, Zhenqiang He, Yujie Ma, Zheng Wang, Ziming Huang, Haihong Cao, Lanyang Xu, Yuheng Zou, Wanyu Wang, Chenpeng Yi, Zhixin Yin, Qirui Wang

**Affiliations:** ^1^Department of Pharmacy, Zhujiang Hospital of Southern Medical University, Guangzhou, China; ^2^Department of Molecular Biology, State Administration of Traditional Chinese Medicine of the People’s Republic of China, School of Traditional Chinese Medicine, Southern Medical University, Guangzhou, China; ^3^State Key Laboratory of Oncology in South China, Department of Neurosurgery/Neuro-Oncology, Collaborative Innovation Center for Cancer Medicine, Sun Yat-sen University Cancer Center, Guangzhou, China; ^4^Breast Surgery Department, Nanyang Central Hospital, Nanyang, China; ^5^School of Basic Medical Science, Southern Medical University, Guangzhou, China

**Keywords:** glioma, tumor microenvironment, tumor associated macrophages, immunotherapy, TAM-targeting therapy

## Abstract

Tumor microenvironment (TME) is the cornerstone of the occurrence, development, invasion and diffusion of the malignant central nerve system (CNS) tumor, glioma. As the largest number of inflammatory cells in glioma TME, tumor associated macrophages (TAMs) and their secreted factors are indispensable to the progression of glioma, which is a well-known immunologically “cold” tumor, including the growth of tumor cells, invasion, migration, angiogenesis, cancer immunosuppression and metabolism. TAMs intimately interface with the treatment failure and poor prognosis of glioma patients, and their density increases with increasing glioma grade. Recently, great progress has been made in TAM-targeting for anti-tumor therapy. According to TAMs’ function in tumorigenesis and progression, the major anti-tumor treatment strategies targeting TAMs are to hinder macrophage recruitment in TME, reduce TAMs viability or remodel TAMs phenotype from M2 to M1. Different approaches offer unique and effective anti-tumor effect by regulating the phagocytosis, polarization and pro-tumor behaviors of macrophages in the therapy of glioma. The present review summarizes the significant characteristics and related mechanisms of TAMs and addresses the related research progress on targeting TAMs in glioma.

## Introduction

Glioma, originated from neuroepithelial tissue, is one of the commonest primary and intractable central nerve system (CNS) malignant tumors with the highest incidence rate, rapidly progressive nature and dismal prognosis (Phillips et al., 2020; Wang et al., 2020). Glioblastoma multiforme [GBM, World Health Organization (WHO) grade IV] is a recalcitrant and incurable kind of glioma with the worst prognosis (Hambardzumyan and Bergers, 2015). Median overall survival (mOS) of GBM patients after comprehensive treatment, covering surgical carcinoma resection, radiation therapy and chemotherapy, chemo-radiotherapy and novel schemes containing tumor-treating fields (TTFields), still hasn’t broken through for 21 months (Pan, 2012; Tan et al., 2020). Complete surgical excision is impractical due to the particular physiological functions of brain tumors and the capacity of GBM to aggressively penetrate surrounding normal brain tissue. In spite of the latest progresses in standardized neurosurgery and the advancement of imaginative multidisciplinary comprehensive treatments against cerebral tumors in perioperative period, there has been exceptionally little enhancement in the treatment of intracranial malignancies. Therefore, huge clinical requirements urge researchers to explore the mechanism of tremendous invasion and recurrence process of glioma, clarify its occurrence and the development of pathogenesis, then find new effective therapies, in which immunologic treatment holds great promise for eradicating glioma (Hervey-Jumper and Berger, 2019; Nejo et al., 2020).

Till now, some seminal immunotherapy, such as immune checkpoint blockade (ICB) treatment (Ito et al., 2019), vaccination therapy (Hilf et al., 2019), chimeric antigen receptor (CAR) T-cell therapy (Bagley et al., 2018) and oncolytic virus therapy (Hua and Wakimoto, 2019), are constantly springing up for GBM treatment. However, these immunologic therapies have not resulted in substantial enhancement in progression-free survival (PFS) or OS (Bell et al., 2018). Pseudoprogression (Galldiks et al., 2017), hyperprogression (Wang Q. et al., 2018), cytokine release syndrome (CRS) (Pang et al., 2018) and high recurrence rate in the front of the operative cavity (Campos et al., 2016) can’t be avoided yet.

For all we know, blood-brain-barrier (BBB), blood-cerebrospinal fluid barrier (BCB) and shortage of the framework for lymphatic drainage serving as a channel that transporting antigen-presenting cells (APC) to the lymph nodes, together engender the CNS immune privilege (Li et al., 2020). The unique physiological advantage of glioma establishes an immune-suppressive and cancer-permissive microenvironment that is featured with high resident and recruited myeloid cell substances, relatively hyporesponsive and exhausted state of tumor infiltrating lymphocyte (TIL), which makes glioma known as immunologically “cold” tumor (D’Alessio et al., 2019). This inherent characteristic makes brain tumors extremely difficult be targeted by T cells. Tumor microenvironment (TME) composing of cancer cells, infiltrating immune cells, endothelial cells, pericytes, fibroblasts, extracellular matrix (ECM) proteins and cytokines, is a complex cellular ecological environment, which evolves together with tumor cells and provides support throughout the transmutation to malignancy (Radin and Tsirka, 2020). Noteworthily, tumor associated macrophages (TAMs), characterized by the highest glucose uptake among TME cells (Reinfeld and Madden, 2021), dominate in number among the inflammatory cells which inhabit the glioma TME and perform a crucial role in neoplasm formation, growth, migration, angiogenesis, immunosuppression, treatment resistance and metabolism (Pan, 2012; Mira et al., 2013; Zhao et al., 2014; Li et al., 2015), and their numbers intimately correlate with glioma grade, suggesting that TAMs may represent an indispensable and pivotal target for brain cancer immunotherapy.

The innate immune system relies heavily on totipotent macrophages, which serve as the initial line of phylactery resisting cancer and infection attack. They are not only the front-line killer cells in infections, but also the maintainers of body homeostasis. They can phagocytize apoptotic cell fragments and secrete growth factors that promote tissue regeneration and angiogenesis, which helps the body constantly renew itself. So macrophages are pluralistic in function (Rothlin and Ghosh, 2020). TAMs are inflammatory infiltrating macrophages settled in the TME that play an integral part in tumor incidence and evolution. Unlike other malignancies, worthy of note is that TAMs in gliomas, generally identified as glioma associated microglia and macrophages (GAMs), originated from not only infiltrating myeloid cells, but also resident microglia. However, the distinction between macrophages and microglia is still being explored, as they can behave separately to different types of CNS injuries (Hambardzumyan et al., 2016). In common, macrophages are broadly classified into two categories depending on their activation status and functions, classic activation of macrophages (M1 macrophages) and alternative activation of macrophages (M2 macrophages) (Najafi et al., 2019). M1 macrophage phenotype can secrete C-X-C chemokine ligand (CXCL)-5, CXCL-9, CXCL-10 and other chemokines, express tumor necrosis factor (TNF)-α, interleukin (IL)-12, IL-2 and other proinflammatory cytokines simultaneously, promote antigen presentation and Th1 activation, and after that play essential roles in anti-tumor protection (Pan, 2012; Zhao et al., 2014). M2 macrophages secrete chemokine (C-C motif) ligand (CCL)-17, CCL-22, CCL-24 and other chemokines, but they are unable to be effectively engaged in antigen presentation. Through the production of inhibitory cytokines such as IL-4, IL-10, and transforming growth factor (TGF)-β, they suppress the immune response, encourage tumor invasion, development and infiltration, enhance angiogenesis, and prevent T cells from having an effective anti-tumor impact (Mira et al., 2013).

**FIGURE 1:**
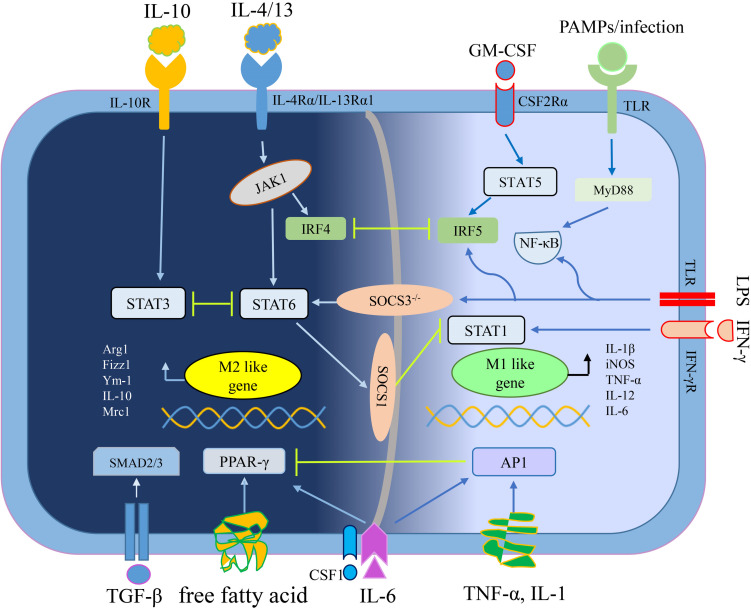
The mechanisms of macrophage polarization. IL-4/13 mediates macrophage polarization toward M2 macrophages depending on STAT6 signaling, whereas IL-10 depending on STAT3 signaling (Lang et al., 2002; Sica and Mantovani, 2012). Free fatty acid or IL-6 plus CSF1 enhance PPAR-γ expression to educate M2 macrophage (Odegaard et al., 2007; Wang et al., 2018b). TGF-β secreted by tumor cells and M2 macrophages promoted M2 polarization of TAMs through Smad3 signaling pathways (Gordon and Martinez, 2010). Interferon-γ (IFN-γ) mediates macrophage polarization toward M1 macrophages relying on STAT1 signaling while other stimuli (like LPS and PAMPs/infections) convert macrophages into M1 phenotype relying on NF-κB signaling (Muraille et al., 2014). GM-CSF mediates macrophage polarization toward M1 macrophages through STAT5 signaling while pro-inflammatory cytokines (including TNF-α, IL-1, and IL-6) depending on AP1 signaling (Liu Y. C. et al., 2014; Wang N. et al., 2014). By the way, the crosstalk between activation of STAT3/STAT6 and STAT1, closely regulates macrophage polarization (Ohmori and Hamilton, 1997). Abbreviations: colony stimulating factor 1 (CSF1), esistin-like-α (Fizz1), Arginase1 (Arg1), chitinase 3-like 3 (Ym1), inducible nitric oxide synthase (iNOS), signal transducer and activator of transcription (STATs), interferon-regulatory factor (IRFs), nuclear factor (NF)-κB, activator protein (AP) 1, peroxisome proliferator-activated receptor (PPAR)-γ, pathogen associated molecular patterns (PAMPs), suppressor of cytokine signaling (SOCS), small mother against decapentaplegic (Smads).

M1, M2 macrophages mainly exist in various stages of the tumor. In the early stage, M1 macrophages mediate anti-tumor effect, while M2 macrophages in the middle-late stage mediate pro-tumor effect. In addition, pro-tumorigenic M2 macrophages and anti-tumorigenic M1 macrophages are in a continuum of polarization states, they can reciprocally transform in the TME, which means there is a dynamic gene expression program (Li et al., 2015). An outline of the polarization mechanism of M1 macrophages and M2 macrophages is illustrated in Figure 1.

## The Performance of TAMs in Glioma Progression

**FIGURE 2:**
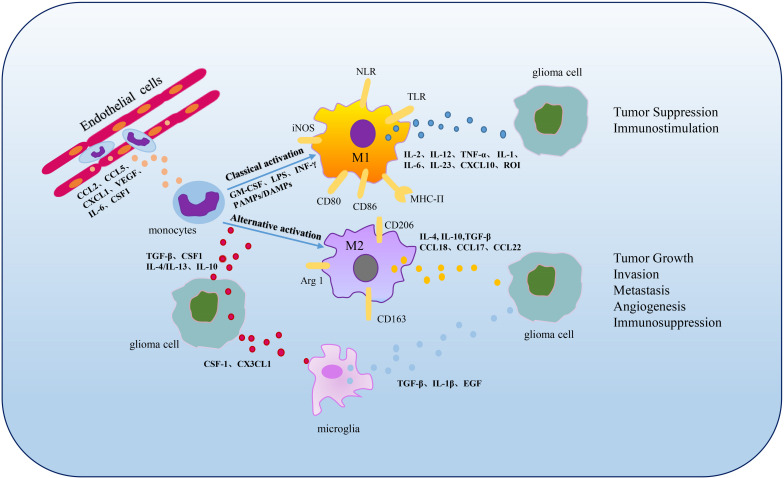
Polarization and functions of GAMs. In the TME, when the BBB is impaired, monocytes infiltrate into tumors and can be differentiated into macrophages stimulated by cytokines involving CSF1, CCL2, CCL5, etc., (Chen and Hambardzumyan, 2018). M1 and M2 phenotypes are the two primary subpopulations of TAMs. M1 phenotype can be activated by cytokines such as GM-CSF, LPS, INF-γ or PAMPs/damage associated molecular patterns (DAMPs) and characterized by secretion of IL-2, IL-12, TNF-α, IL-1, IL-6, IL-23, CXCL10 and ROI, functioning in tumor suppression and immunostimulation (Lee et al., 2020; Xu et al., 2020). M2 phenotype can be activated by cytokines including TGF-β, CSF1, IL-4, IL-13 or IL-10 and secrete large amounts of IL-4, IL-10, TGF-β, CCL18, CCL17 and CCL22, contributing to tumor growth, invasion, metastasis, angiogenesis and immunosuppression (Xu et al., 2020). Microglia, the resident macrophages in CNS, can be activated by CSF1 and CX3CL1 secreted by glioma cells and up-regulate TGF-β, IL-1β, EGF expression to promote glioma development and migration (Marchesi et al., 2010; Hambardzumyan et al., 2016).

As a key segment of TME and the initial response of the host immune system’s defense, TAMs are extremely powerful in tumor expansion and progress, including tumor genesis, invasion, migration, angiogenesis, immunosuppression and metabolism (Figure 2).

### Tumor Growth

Both TAM master regulators and their effector genes are frequently associated with tumor growth and strongly linked to poor clinical outcomes. TAMs secrete effector cytokines such as epidermal growth factor (EGF), platelet derived growth factor (PDGF), basic fibroblast growth factor (bFGF), hepatocyte growth factor (HGF) and TGF-β, which can promote tumor cells proliferation and survival, based on a huge number of animal studies (Kennedy et al., 2013; Caux et al., 2016; Liu et al., 2018; Wang C. et al., 2018). McFarland et al. (2013) found TNF-α could stimulate IL-6 production and trigger NF-κB and STAT3 pathways to enhance glioma cells proliferation both *in vivo* and *in vitro*. Yan et al. (2015) and other researchers have verified that M2 macrophages residing in the tumor tissue showed high expression of Tim-3 gene. Knocking out Tim-3 in M2 macrophages inhibited the growth rate of cancerous cells. In glioma, glial TIM-3’s level is diminished and several inflammation-related genes exhibit a lesser increase in Tim-3^mut^ glia compared to WT glia when exposed to glioma conditioned medium (GCM) (Kim et al., 2020). Normal macrophages synthesize NO by using iNOS and L-arginine as substrates, thereby acting as a cytotoxic effect. However, TAMs expressing Arg1 are strengthened by IL-4/13 signaling *via* the blocking effect of transcription factor STAT6, thus to reduce NO synthesis, promote the production of polyamines, and advance tumor progression (Pesce et al., 2009; Mantovani et al., 2013; Pires-Afonso et al., 2020).

### Tumor Invasion and Migration

Glioblastoma multiforme has the characteristics of lethal intracranial invasion and rare extracranial metastases, and is often locally invasive (Rossi et al., 2020). Inside the brain, it may be due to the existence of BBB and the loss of a lymphatic drainage system, extra-neural metastases of glioma are rare, but still may occur in the later course of GBM. GBM metastases usually include the regional lymph nodes, lungs, and pleural cavity, as well as the bone and liver on rare occasions (Beauchesne, 2011). The degradation and damage of the basilar membrane of tumor vascular endothelial cells are the main factors leading to tumor cell infiltration and metastasis. In the TME, TAMs can disrupt cell-cell communication, destroy the basilar membrane by secreting serine proteases, matrix metalloproteinases (MMPs), cathepsins, etc., as well as promote endothelial cell viability, thus facilitating invasion and metastasis of tumors (Das et al., 2011; Yang et al., 2017; Quintero-Fabián et al., 2019). For example, MMPs are observed in a collection of malignancies, involving glioma, gastrointestinal stromal tumors, pancreatic cancer, breast cancer and other malignant tumors, and have been associated with tumor infiltration, metastasis and prognosis (Leifler et al., 2013; Liou et al., 2013; Wang C. et al., 2014). Growth factors, interleukin and extracellular matrix constituents (e.g., TGF-β, IL-6, PDGF, FGF and MMPs) generated by TAMs may cause tumor cells to go through GMT (glial-to-mesenchymal), as well as ease divergent invasions. Zhang et al. (2012) co-cultured CCL2^+^ U87 glioma cells with microglia, and stated that microglia with CC chemokine receptor (CCR) 2 highly expressed IL-6, thus enhancing the aggressiveness of glioma cells.

### Tumor Angiogenesis

During the development of the tumor, the newly generated blood arteries sustain oxygen and nutrition to tumor, authorizing it to grow and invade while macrophages secrete angiogenesis-promoting growth substances such as vascular epithelial growth factor (VEGF)-family members, CSF1, WNT family members etc., speeding the degradation of the perivascular extracellular matrix to assist tumor angiogenesis (De Palma et al., 2017). Studies have shown that TAMs infiltrated in tumor tissue accumulate in hypoxic regions with less micro vessels. Hypoxic niche induces TAMs to highly express numerous angiogenic and cytokines as well as a large number of angiogenic regulatory enzymes, such as VEGF, PDGF, fibroblast growth factor (FGF1, FGF2), placental growth factor (PIGF), HGF, (bFGF, IL-1, IL-8, MMP-9, MMP-2, TNF-α, urokinase plasminogen activator (uPA), adrenomedullin (ADM) etc., to spur tumor angiogenesis (Peng et al., 2005; Qian and Pollard, 2010; Wang et al., 2011; Zhang et al., 2011; Meng et al., 2012). In glioma, CD163^+^ or tunica interna endothelial cell kinase 2 (Tie-2) positive TAMs are identified in parenchymal and perivascular niche. Zhu et al. (2017) reviewed key roles of TAMs in angiogenesis through autocrine and paracrine. Take inflammatory cytokines for example, IL-1β synthesis by COX2^+^ TAMs stimulated by GBM-derived C-reactive protein (CRP) can enhance the expression of pro-angiogenic factors in endothelial cells. TAMs can secret IL-6, which promotes GMT of high-grade gliomas. Moreover, IL-6 can recruit endothelial progenitor cells and promote vasculogenesis as well. Notably, it is interesting that in the previous study (Wang et al., 2018b), we found glioma endothelial cells can also secrete IL-6 and M-CSF to induce alternative macrophage activation through PPAR-γ/HIF 2α pathway. Thus, vascular niche and TAMs regulate each other positively, together promoting the formation and deterioration of glioma immunosuppressive microenvironment.

TAMs can also benefit lymph-angiogenesis by the expression of VEGF-C, VEGF-D, VEGFR3, and other diverse components involved in regulating this process. Kerjaschki (2005) proposed hypothesis that there may be two ways to induce lymph-angiogenesis by macrophages in TME. One is the direct *trans-*differentiation of macrophages into lymphatic endothelial cells, and the other is the stimulation of macrophages on the division and multiplication of lymphatic endothelial cells. Jung et al. (2016) discovered that multicellular signaling circuits were involved in flowing out sphingosine 1-phosphate (S1P) by dying human MCF-7 breast cancer cells, stimulating M2 macrophages to release lipocalin 2 (LCN2), and inspiring lymphatic endothelial cells to deliver lymph-angiogenic factor VEGF-C, thus promoting the growth of lymphatic vessels in the TME. Although there’s little referring to GAMs, with the discovery of central nervous system lymphatic vessels, the relationship between GAMs and lymph-angiogenesis in gliomas may be a challenging research topic in the future (Louveau et al., 2015).

### Immunosuppression in the TME

**FIGURE 3:**
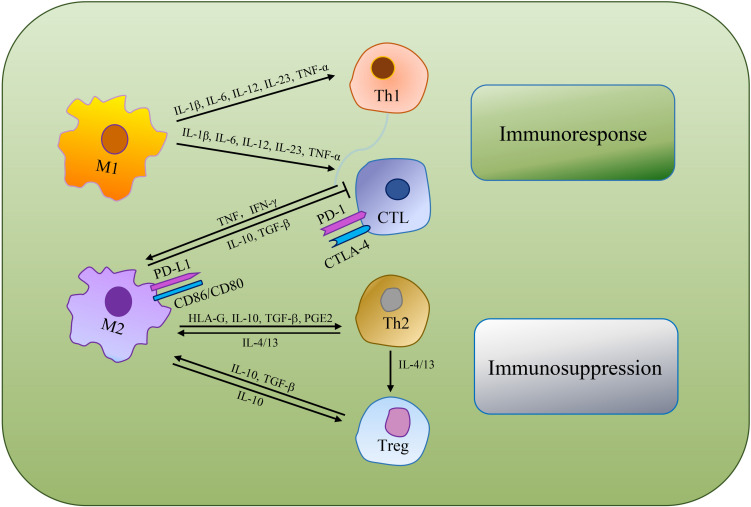
The mechanisms of TAMs regulating tumoricidal T cells. TAMs can directly suppress CTLs or indirectly regulate the differentiation of Th cells and Tregs to make an immunosuppressive TME.

Immunosuppression is a prerequisite for tumor formation and growth. Macrophages are qualified of offering antigens to effector cells to turn on anti-tumor functions of T cells and NK cells. Whereas, alternative activated microglia/macrophages and inactivated T-killer cells, resulted in characteristic immunosuppressive TME. Single-cell transcriptomic atlas discovered inter-/intra-tumor heterogeneity, as well as enlarged depleted T cells, Tregs and other innate immune cells in GBM (Fu et al., 2020). Chen and Hambardzumyan (2018) reviewed the immunosuppressive functions of GAMs and mechanisms of GAMs inhibiting the functions of cytotoxic T cells (CTLs). As GAMs are remarkably plastic, M1 macrophages produce abundant pro-inflammatory cytokines (e.g., IL-1β, IL-6, IL-12, IL-23, and TNF-α) that support NK cells and Th1 cells mediate anti-tumor activity, while also promote CD4^+^ T cells to release cytotoxic products to help achieve anti-tumor effect (Jiang et al., 2014; Shapouri-Moghaddam et al., 2018; Orecchioni et al., 2019). M2 macrophages can secrete human leukocyte antigen-G (HLA-G), IL-10, TGF-β, PGE2 and other immunosuppressive molecules, directly inhibiting immune response (Wu et al., 2010). IL-10 plays a negative role in regulating IL-12, which has the capacity to support Th2 cells differentiation, release notable quantities of IL-4 and IL-13 to convince regulatory T cells (Tregs) amplification and expedite TAMs development toward a pro-tumor phenotype. Besides, M2 macrophages can suppress CTLs *via* TGF-β and IL-10 production (van Dalen et al., 2018; DeNardo and Ruffell, 2019; Figure 3).

In animal experiments, IL-10 suppressed the immune response by inhibiting the NF-κB signaling route (Yang et al., 2014). Moreover, cancerous cells can secrete CSF1, which can bind to colony stimulating factor 1 receptor (CSF1R, also named as FMS) on macrophages, activating the downstream signaling pathway in charge of TAMs polarization toward the immunosuppressive M2 phenotype (Ramesh et al., 2019). TAMs and myeloid-derived suppressor cells (MDSCs) are powerful immunosuppressors in the glioma TME (Zinnhardt et al., 2020). STAT3 presents a prominent function in monitoring MDSC’s differentiation into TAMs, together contributing to an immunosuppressive TME (Kumar et al., 2016; Pinton et al., 2020). Programmed death receptor-1 (PD-1) is an inhibitory immunological checkpoint on T cells which was originally obtained by subtracting hybridization techniques in apoptotic T cell hybridomas. Reacting with its ligand, PD-1 can regulate the body’s immune system reaction. Programmed cell death ligand 1 (PD-L1), which aids tumor’s immune evasion, is described on both tumor cells and TAMs in a collection of malignancies including glioma. PD-L1 on macrophages can lead to restraint of T cell activation (DeNardo and Ruffell, 2019), phagocytosis and tumor immunity (Hutchinson, 2017). TAM-derived PD-1 expression is negatively related to the macrophage phagocytosis against tumor cells. An excess of reactive oxygen species (ROS) and peroxynitrite can be produced to inhibit T cells-regulated anti-tumor responses (Kortylewski et al., 2005; Du et al., 2012). PD-1 blockade *in vivo* ameliorated macrophage phagocytosis against cancer cells, diminished tumor formation, and prolonged the survival of macrophage-dependent mouse models, which confirmed that PD-1/PD-L1 blockade could interfere with the tumor formation by direct action on macrophages (Gordon et al., 2017). The pre-existing tumor infiltration of CD8-positive cytotoxic T cells is a promising marker of ICB treatment response. By blocking Fcγ receptors (FcγRs) before the administration of anti-programmed cell death protein 1 monoclonal antibodies (aPD-1 mAb), time for aPD-1 mAb binding to tumor-infiltrating CD8^+^ T cells perpetuated, which advanced tumor regression in mice treated with immunotherapy (Arlauckas and Garris, 2017).

### The Crosstalk Between Glioma Stem Cells and TAMs

Cancer stem cells (CSCs), noted as tumor-propagating cells or tumor-initiating cells as well, refer to a distinct subset of cells holding many stem-like aspects such as tumorigenesis, self-renewal and multi-directional differentiation (Nassar and Blanpain, 2016). CSCs have been considered as a critical driver of tumor initiation. Detailed surveys have shown that TAMs can regulate the performances of CSCs in diverse carcinomas. Shi et al. (2017) reported that TAMs promoted GBM malignant growth by secreting pleiotrophin (PTN) in PTN–PTPRZ1 paracrine signaling to stimulate glioma stem cells (GSCs) through its receptor PTPRZ1. Ye et al. (2012) revealed that TAMs enhanced the invasiveness of GSCs by expressing TGF-β1 to augment MMP-9 generation by stem-like cells in glioma. Zhang et al. (2017) labeled CD163 and CSC-related proteins by immunohistochemistry (IHC) and found that CD163^+^ TAMs were excessive in gastric cancer (GC) tissues with invasive tendencies, which was an independent worse prognostic factor in GC. What’s more, over-expression of CD163 in TAMs was linked to high levels of CSC markers, implying that CSCs in GC may act as a promoter for tumor progression and aggressiveness, and polarized CD163^+^ TAMs may aid in the maintenance of CSCs in GC tumors (Zhang et al., 2017). According to Yang et al.’s research, TAMs can promote phosphorylation of STAT3 by activating EGF signaling in mouse breast cancer cells and induce SOX-2 expression which maintains tumor cell CSCs phenotype, and identified a novel role of TAMs in breast CSC regulation (Yang et al., 2013). However, the study of the relationship between macrophages and CSCs is still in its infancy so far and requires further study.

### Macrophages Cell Fusion

Cell fusion is a unique cell biological phenomenon, referring to the process of re-integration of two or more cells into one cell, which is crucial in the body development and tissue repair. Cell fusion can be observed at various developmental stages, such as fertilization (sperm and egg fusion), bone development (macrophage differentiation into osteoclasts) and immune responses (macrophages fuse into megakaryocytes) (Bastida-Ruiz et al., 2016).

In the process of tumor development, cell fusion also plays an important part. As early as 1911, the German pathologist Otto Aichel proposed the cell fusion hypothesis that cancer cells can be fused with white blood cells to form malignant hybrid cells, leading to tumor development. Current studies have proved that tumor cells can be fused with a sort of bodily cells, covering interstitial cells, epithelial cells and endothelial cells. After fusion with normal leukocytes with low metastatic ability, the metastasis ability of tumor cell is enhanced (Bastida-Ruiz et al., 2016). When M2 macrophages and MCF-7 breast cancer cells were co-cultured, their fusion produced metastatic hybrids with maternal cytogenetic and phenotypic characteristics. By hybridizing with macrophages, MCF-7 cells gain CD163 and CD45 double markers, suggesting that cell fusion might result in tumor clonal explosion and heterogeneity (Shabo et al., 2015).

### Metabolic Changes of TAMs in Glioma

In the early stage of tumor, TAMs mediate anti-tumor effect mainly through antioxidant function, displaying an active glycolytic metabolism. Within the progress of the tumor, malignant tumor cells constantly adjust their metabolic patterns to obtain sufficient nutrition and induce increased fatty acid oxidation of TAMs to promote the tumor development (Boscá et al., 2015). M1 macrophages have an enormous amount of aerobic glycolysis activity and can produce ROS to kill pathogens. M2 macrophages release substantial VEGF-A and IL-10 to support tumor cells proliferation mainly by oxidative phosphorylation. Under the stimulation of hypoxia and lactic acid, TAMs are easy to produce cytokines like IL-6, CCL5 and CCL18. CCL5 and CCL18 up-regulate the activity of various glycolysis factors, including lactate dehydrogenase A (LDHA) and glucose-6-phosphate dehydrogenase (G6PD), which can advance glycolysis in tumor cells, lead to accumulation of lactic acid in TME and restrain the immune system’s anti-tumor reaction (Colegio et al., 2014). M2 macrophages can serve tumor cells’ evasion of immune detection by overexpressing Arg1 and catalyzing tryptophan metabolites, consuming amino acids in TME and challenging for nutrients with T and NK cells (Liu et al., 2017). Notably, for the latest survey data on myeloid cells, especially TAMs, showed the highest glucose uptake across a range of cancer models while cancer cells had the greatest capacity to take up glutamine. Cell-intrinsic programs derived cell-selective acquisition of glucose and glutamine by immune and cancer cells, respectively. Thus, a new therapy strategy could be exploited (Reinfeld and Madden, 2021).

## Therapy Of Glioma Targeting TAMs

The leading anti-tumor treatment strategies targeting TAMs are updated and currently contain: (1) restricting macrophages entrance into TME or reducing TAMs viability; (2) reeducating TAMs phenotype to M1 phenotype exerting anti-cancer effects; (3) enhancing the phagocytosis and antigen presenting ability of macrophages or macrophage-mediated anti-cancer function. Current advances in TAM-targeting therapies are listed below (see Table 1 for summary).

**TABLE 1:** Macrophages targeting therapies in cancers.

Substance	Target	Mechanisms	Tumor type / study type	Side effects	References
Nivolumab	*PD1*	Enhancing cytotoxic activity and phagocytosis to tumor cells	Glioma:Phase 3	Hypertension, fatigue	Reardon et al., 2020
WP1066	*STAT3*	Inducing costimulatory molecules expression and increasing immune-stimulatory cytokines production and T-cell proliferation	Glioma:preclinical trial melanoma:Phase 1	Unknown	Hussain et al., 2007
Anti-CD47 antibody	*CD47*	Enhancing cytotoxic activity and phagocytosis to tumor cells	Glioma:preclinical trial	Unknown	Hayat et al., 2020
OLA-PEG, NOX-A12	*SDF-1*	Inhibiting the recruitment of TAMs	Glioma:preclinical trial	Unknown	Deng et al., 2017
POL5551	*CXCR4*	Inhibiting hypoxia-induced glioma cell migration and reducing tumor-initiating GSCs and MGCs populations	Glioma:preclinical trial	Unknown	Gagner et al., 2017
ACF	*TGF-*α	Blocking hypoxia-induced POSTN over-expression by TGF-α through the RTK/PI3K pathway to inhibit the recruitment of macrophages.	Glioma:preclinical trial	Unknown	Guo et al., 2016
BLZ945	*CSF1R*	Inhibitor of CSF1R, preventing glioma progression	Glioma:preclinical trial	Unknown	Pyonteck et al., 2013
GK921	*TGM2*	Triggering MES transdifferentiation of GSCs	Glioma:preclinical trial	Unknown	Yin et al., 2016
STX-0119	*STAT3*	The STAT3 inhibitor, promoting TILs accumulation	Glioma:preclinical trial	Unknown	Akiyama et al., 2017
FLA-16	*CYP4A*	Inhibiting angiogenesis	Glioma:preclinical trial	Unknown	Wang et al., 2017
ISL	*Akt- FGF-2/TGF-*β*/VEGF*	Inhibiting angiogenesis	Glioma:preclinical trial	Unknown	Wang et al., 2019
Flavonoid CH625	*CYP4X1*	Inhibiting angiogenesis	Glioma:preclinical trial	Unknown	Wang C. et al., 2018
TrLp	*p53*	Increasing the TAMs-depletion capability	Glioma:preclinical trial	Unknown	Mukherjee et al., 2018
miR-124	*STAT3*	Inhibiting the STAT3 pathway and reversing T-cell proliferation immunosuppression	Glioma:preclinical trial	Unknown	Wei et al., 2013
Trabectedin	*CCL2-CCR2 axis; caspase-8*	Inhibition of macrophage differentiation and cytokine production and improvement of the TAMs-depletion capability	Metastatic soft-tissue sarcomas:Phase 1/2 Breast cancer:Phase 2	Neutropenia, hepatic (transaminitis), fatigue, nausea, vomiting, constipation	Martin-Broto et al., 2020
PLX3397	*CSF1R*	Inducing gene expression of M2-like macrophages	Tenosynovial giant-cell tumors:Phase 1/2	Changes in hair color, fatigue, nausea, dysgeusia, periorbital edema, diarrhea, hyponatremia, etc.	Tap et al., 2015
AZD1480	*ICAM-1 and pSTAT3*	Suppressing macrophage infiltration into the tumor site	Lung cancer:Phase I	Dizziness, ataxia, hallucinations, and anxiety	Piao et al., 2017
Ablation of NRP-1	*NRP-1*	Inhibition of Angiogenesis and interfering the interaction of TAMs with the TME	Breast cancer:preclinical trial	Unknown	Kawaguchi et al., 2017
TMP195	*CSF1 and CSF2*	Regulating the polarization of TAMs	Breast cancer:preclinical trial	Unknown	Guerriero et al., 2017
Leg-3	*Legumain*	Activating the ablation of TAMs	Breast cancer:preclinical trial	Unknown	Lin et al., 2013
BLIMP1	*CCL8*	Suppressing CCL8 expression to modulate host defenses	*L. monocytogenes* infection:preclinical trial	Unknown	Severa et al., 2014

### Inhibiting Macrophage Recruitment in TME or Reducing TAMs Viability

#### Reducing the Recruitment/Activation of TAMs

CD70, a TNF family member declared solely on tumor cells and not on macrophages, promotes tumor aggressiveness and immunosuppression by recruiting and activating TAMs (Bowman et al., 1994; Wischhusen et al., 2002). Ge et al. (2017) found that CD70 expression was strongly linked to the incidence of CD163^+^ macrophages in GBM, indicating CD70 functioned by attracting TAMs to the tumor sites. It has been demonstrated that CD70-targeting therapy showed a profound anti-tumor effect in preclinical studies in orthotopic model of GBM, providing a ray of light for CD70^+^ GBM patients. TAMs depend on CSF1 for proliferation, differentiation, survival and recruitment (Ramachandran et al., 2017; Peranzoni et al., 2018). One study of CSF1R restraint showed that TAMs were not depleted but M2 markers reduced in surviving TAMs in line with enhanced survival in GBM patients. The CSF1/CSF1R axis has gained a lot attention and clinical development is ongoing. BLZ945, as the inhibitor of CSF1R, prevented glioma progression and improved survival in animal experiments by perturbing macrophage survival (Pyonteck et al., 2013). What’s more, a number of small molecules and mAbs targeting CSF1/CSF1R axis are being tested in melanoma, prostate cancer and other solid tumors (Cannarile et al., 2017).

#### Suppression of Macrophage Migration Into Tumors

ICAM-1, which is activated by pSTAT3 in hypoxic sites, facilitates glioma cell passage and carcinoma evolution. ICAM-1 knockdown decreased macrophages recruitment into carcinomas *in vivo*. *In vitro*, anti-ICAM-1 antibody inhibited tumor cell migration to macrophages (Yin et al., 2016). Besides, ICAM-1 was linked to the GAMs embroiled in resistance of glioma to antiangiogenic treatment. These findings suggested that ICAM-1 knockdown may be a helpful strategy for overcoming the resistance of GBM to antiangiogenic therapy (Piao et al., 2017). Blocking PD-1/PD-L1 has been announced directly effect on macrophages (Gordon et al., 2017). Monoclonal antibodies that point the PD-1/PD-L1 axis have been testified to be remarkably practical in clinical tumor cases. Pembrolizumab and nivolumab (NIVO, checkpoint inhibitor for PD-1) were authorized by the Food and Drug Administration (FDA) for melanoma and non-small cell lung cancer (NSCLC) treatment in 2014 and 2015 subsequently (Xue et al., 2017). The first large-scale phase III trial of NIVO in GBM patients (CheckMate 143, NCT02017717, initiated in January 2014 and data cutoff of January 20, 2017) showed a median follow-up of total 369 patients randomized to NIVO or bevacizumab. The results showed mOS was comparable and the safety profile of nivolumab was consistent with other tumor types (Reardon et al., 2020).

#### Increasing TAMs-Depletion Capability

Blood-brain-barrier, Blood-Brain tumor Barrier (BBTB), vasculogenic mimicry (VM) channels and TAMs are all natural restrictions of the therapeutic drugs for glioma. *In vitro* and *in vivo*, a synergistic anti-glioma activity was discovered when Lycobetaine (LBT) and octreotide (OCT) were combined. LBT and OCT co-loaded liposomes (LPs) showed outstanding anti-cancer and anti-angiogenesis efficacy. nRGD has the potential to improve selective glioma cell targeting efficiency, intra-glioma drug delivery and TAMs reduction. As a result, nRGD modified LPs proved to be a bright and flexible medicine delivery system for glioma treatment (Chen et al., 2017). Of note, latest investigation has indicated that macrophages were decisive in internalizing designed nanocarriers before being polarized or reprogrammed (Ye et al., 2019). Moreover, various liposomes have been designed to deplete or re-educate TAMs by targeting them through cell-specific surface receptors. Mukherjee et al. (2018) pointed out that liposomal TriCurin (TrLp), a mixture of curcumin, epicatechin gallate, and resveratrol, boosted activated p53 in GL261 cells, repolarized TAMs and eliminated glioblastoma cells as well as GSCs by triggering an apoptosis cascade.

#### Targeting Chemokines in Hypoxia-Niche of Glioma

Macrophage/microglial cells heavily infiltrate into most human glioma tumors, occupying up to 30% of the tumor’s bulk. TAMs are attracted to the glioma community and secrete a variety of growth factors and cytokines in reaction to cancer cells stimuli and help tumor growth, survival, and relocation in a regurgitation-feeding way (Hambardzumyan et al., 2016).

CXCR4, the receptor of stromal cell-derived factor (SDF)-1α, can be induced to overexpress on invading tumor, macrophages/microglia and GSCs in hypoxia-niche, and is a therapeutic target for patients suffering from GBM (Wang et al., 2012). Deng et al. (2017) demonstrated that SDF-1 blockade with olaptesed pegol (OLA-PEG, NOX-A12) impeded TAMs recruitment by VEGF blockage in GBM. POL5551, a potent CXCR4 antagonists, could decrease the populations of tumor-initiating GSCs and GAMs, as well as lessen hypoxia-induced uncontrolled multiplication and invasion of glioma (Gagner et al., 2017). Guo et al. (2016) discovered that the hypoxia-inducible factors (HIFs) inhibitor, acriflavine (ACF), blocked the over-expression of hypoxia-inducible POSTN by virtue of TGF-α through RTK/PI3K pathway.

### Reeducating TAMs to M1 Macrophages

#### Minimizing the Immunosuppressive M2 Macrophages or Blocking the Signaling Pathways Displaying to CSCs

There was direct evidence that the existence of CSCs is the real driving force behind proceeded recurrence of brain tumors following chemotherapy (Hemmati et al., 2003). These cells have strong DNA repair, multipotency, self-renewal and tumor recapitulation ability *in vivo*. Even if the proportion of these cells in primary tumor cells is constrained, they have the capacity to fortify regrowth, and attack normal surrounding brain tissues to form local metastasis (Ortensi et al., 2013). MicroRNAs (miRNAs) are short non-coding RNAs that act *via* mRNA degradation, deadenylation or translational repression. Studies uncovered that miRNAs were fundamental for various pathophysiological processes, including cell proliferation, differentiation and apoptosis (Saliminejad et al., 2019; Zhang L. et al., 2019). Liu Y. et al. (2019) identified that miR-340-5p over-expression restrained TAMs thickness, M2 macrophages activation and tumorigenesis of glioma *in vivo*.

CSCs tend to regulate macrophage polarization by activating STAT3. TGF-β1 signaling allows M2 macrophages originating from peripheral blood monocytes to improve CSC functions. Moreover, siRNA-mediated STAT3 silencing could cut off CSC chemo-receptivity and migratory capacities. Suppressing immunosuppressive macrophages or disturbing the messages they send to CSCs may have unexpected effect in GBM combination therapies (Nusblat et al., 2017).

As a molecular chain, the PTN-PTPRZ1 paracrine signaling regulated M2 macrophages supportive ability on GBM destructive development and GSC maintenance. Disrupting PTN-PTPRZ1 signaling could effectively inhibit GSC-driven tumor development, and this result meant that targeting PTN-PTPRZ1 signaling therapy could enhance treatment for GBM and other malignant tumors (Shi et al., 2017).

As nanomedicine advances, sophisticated nanocarriers developed for specific receptors on TAMs are a very promising TAM-targeting technique. Ovais et al. (2019) reviewed the existing cancer immuno-nanomedicines, which mainly affect the survival of M2-like TAMs or their signaling pathways, prevent macrophages from recruiting to tumor sites and retrain M2-like macrophages to M1-like phenotype. One subject demonstrated that in a GBM model, transcribed mRNA encoding M1-polarizing transcription factors could be addressed by a specific nanocarrier to reprogram TAMs (Zhang F. et al., 2019).

#### Inducing M2-Like Gene Expression Changes in TAMs

TAMs could be depolarized from the initial M2-like state after being exposed to CSF1R inhibitors such as PLX3397, thus effectively impairing TAMs’ pro-tumorigenic activities and further presumably led to reduction in tumor development or regression *in vivo* (Tap et al., 2015). Compared with PLX3397 monotherapy, it was found that combining PLX3397 with Dovitinib or Vatalanib further diminished M2-like genes expression, including Stab1, Irf4 and Ccl22 (Yan et al., 2017). Wei et al. (2013) identified a key candidate gene for regulating STAT3 signaling pathways, miR-124. STAT3 pathway could be blocked by upregulating miR-124 in GSCs. miR-124 inhibited T-cell proliferation and forkhead box P3 positive (Foxp3^+^) Tregs activation through reversing GSCs-mediated immunosuppression. Zhang F. et al. (2019) introduced a specific nanocarrier, which could transport *in vitro*-transcribed mRNA to reprogram TAMs by encoding M1-polarizing transcription factors. By encoding IRF5 together with its activating kinase IKKβ, TAMs were reprogrammed to trigger anti-tumor response and facilitated tumor cell death after infused of nanoparticles formulated with mRNAs to reverse their immunosuppressive, tumor-supporting state.

### Enhancing the Phagocytosis and Antigen Presenting Ability of Macrophages or Macrophage-Mediated Anti-cancer Function

#### Targeting Special Molecules Expressed on Macrophages

Mer tyrosine kinase (MerTK), expressed on GAMs, is a receptor tyrosine kinase (RTK) that activates efferocytosis (specific type of phagocytosis) and suppresses innate immune responses. Inhibition of MerTK combined with external beam radiotherapy (XRT) has been proved to be an effective way in treating a subset of GBMs (Wu et al., 2018). CCL2 (also named MCP-1), which is released by tumor cells, is a chemotactic factor that recruits CCL2R^+^ monocytes, T cells and NK cells. Both antibodies against CCL2 as well as inhibitors of CCL2R have shown evidence of reducing cancer progression (Li et al., 2013; Cassetta and Pollard, 2018). Allavena et al. (2005) and Martin-Broto et al. (2020) detailed an imaginative anti-tumor item, yondelis (trabectedin), which inhibited macrophage differentiation and cytokine yield in metastatic soft-tissue sarcoma, ovarian and breast adenocarcinoma. Takenaka et al. (2019) reported that the production of kynurenine by glioma activated aryl hydrocarbon receptor (AHR), which drove TAMs enrollment in response to CCL2 and suppressed NF-κB activation in TAMs to modulate their functions.

#### Targeting Macrophages/Microglia-Derived Cytokines

Yin et al. (2017) reported that through NF-κB activation, macrophages/microglia-derived cytokines heightened transglutaminase 2 (TGM2, an inducible transamidating acyltransferase) expression. TGM2 is increased within the perinecrotic zone of GBM and promotes mesenchymal (MES) *trans-*differentiation in GSCs by controlling master transcription factors (TF) involving C/EBPβ, TAZ, and STAT3. It has been illustrated that TGM2 was a crucial switch protein in MES *trans-*differentiation triggered by necrosis, so it is a promising treatment target for MES GBM.

In the process of glioblastoma, TAMs are recruited and induced by cancer cells to enhance tumor development (Mantovani et al., 2002), that is, tumor cells inhibit the anti-tumor immunological reaction of TAMs and urge TAMs to secret IL-6, VEGF, MMPs, IL-10 and TGF-β1 to favor tumor cell expansion, angiogenesis, matrix degradation and invasion (Joseph et al., 2015). TGF-β inducible protein (TGFBI) is one of many transduction regulatory molecules in TGF-β signal pathways relying on classical SMAD pathways and non-SMAD pathways (Rodríguez-García et al., 2017; Deng et al., 2018; Guo et al., 2018). One study discovered that there was a parallel relationship between the distribution of TGFBI and TAMs based on the inspection of tumor Genome Map Project (TCGA), Chinese Glioma Genome Map Project (CGGA), brain tumor Molecular Database (REMBRANDT) and Oncomine Database (Li and Graeber, 2012). Zhang et al. (2020) revealed that CCL8 (also referred to as MCP-2) delivered by TAMs promoted invasion and GBM cells stemness by mobilizing the phosphorylation of ERK1/2 in GBM cells. Severa et al. (2014) recorded the transcriptional repressor B lymphocyte-induced maturation protein 1 (BLIMP1) conditional knockout (CKO) mice showed stronger levels of circulating CCL8, uncovering that BLIMP1 performed a fundamental task in modifying host defense mechanisms by decreasing CCL8-expression.

#### Promotion of Tumor-Infiltrating Lymphocyte Accumulation and T Cell Activation

STAT3 participates in many of the widely active carcinogenic signaling as well as the transcriptional modulation of different tumor-promoting factors. STX-0119, the STAT3 inhibitor, demonstrates anti-tumor activity occurring through promoting TILs accumulation predominantly in the clonal expansion of CD8-postitive T cells and macrophages at TMZ-resistant U87 glioma tumor-site and humanized MHC-double knockout mouse (dKO-NOG) system, which has the potential to be a critical tool for assessing the impact of STAT3 inhibitors on human tumors (Akiyama et al., 2017). WP1066, a small-molecule STAT3 pathway inhibitor, increased the secretion of cytokines such as IL-2, IL-4, IL-12, IL-15, and T-cell proliferation by turning on co-stimulatory molecules expression on microglia and peripheral macrophages (Hussain et al., 2007).

#### Angiogenesis Inhibition in Glioma

VEGF and TGF-β are pivotal factors in glioma angiogenesis. Conditional medium collected from TAMs and EPCs that dealt with a flavonoid (FLA-16) caused pericyte cells relocation thus decreasing endothelial cells proliferation and migration, which could be shifted by cytochrome P450 (CYP) 4A (CYP4A) over-expression or exogenous supplement of 20-hydroxyeicosatetraenoic acid (20-HETE), VEGF or TGF-β. Moreover, FLA-16 inhibited crosstalk between TAM and EPC during angiogenesis. Repression of CYP4A by FLA-16 prolonged life and normalized tumor vasculature in GBM by hindering TAMs and EPC-derived VEGF and TGF-β through PI3K/Akt signaling (Wang et al., 2017). In addition, Arachidonic acid (AA) metabolic enzymes, including microsomal prostaglandin E synthase-1 (mPGES-1), cyclooxygenase-2 (COX-2) and CYP4A11, are vital to glioma angiogenesis. Wang et al. (2019) detailed that through the ceRNA effect of miR-194-5p and lncRNA NEAT1, flavonoid isoliquiritigenin (ISL) hindered the Akt/FGF-2/TGF-β/VEGF angiogenic signaling to reprogram AA metabolism mediated by COX-2, mPGES-1 and CYP4A in glioma. Wang C. et al. (2018) found that flavonoid CH625 (a flavonoid, 3-sulfanyl-1-triazene) inhibition of CYP4X1 in TAMs normalized tumor vasculature in glioma *via* cannabinoid receptors (CB) 2/EGFR-STAT3 axis, and this effect could be reversed by CYP4X1 and STAT3 over-expression, as well as external stimulants, such as 14,15-epoxyeicosatrienoic acid-ethanolamide (14,15-EET-EA), VEGF, TGF-β, EGF, and AM630 (the CB2 inhibitor) and so on. Neuropilin-1 (NRP-1), a transmembrane receptor, can amplify pro-angiogenic signaling in the TME. NRP-1 positive macrophages play an imperative role in antibody-mediated immune responses to fight cancer. Evidence showed that knockdown of NRP-1 on macrophages suppressed lymphocyte infiltration in TME. NRP-1 down-regulation on macrophages modulated tumoricidal function mediated by antibodies. *In vivo* studies with a human breast cancer xenograft model revealed that NRP-1 expression on macrophages was necessary for antibody-based tumoricidal activity and infiltration of CD4^+^ T cell into tumor locations (Kawaguchi et al., 2017). Several experiments demonstrated that NRP-1 signaling in GAMs were essential for their intertwined links with the surrounding glioma microenvironment. Ablation of NRP-1 from GAMs exhibited less vascularity and slowed tumor progression (Miyauchi et al., 2016).

## Potential Targets for TAMs in Other Tumors

Numerous investigations have described that TAMs are intimately linked to tumorigenesis. Hence, TAM-targeting immunotherapy shows great potential and promising prospects in tumor therapy. The potential targets of TAMs recently found in glioma and other cancer studies are listed below (Table 1), including TAMs activation inhibitor or direct targets for TAMs, in order to provide some inspiration in the subsequent treatment of glioma.

### Enhancing Cytotoxic Activity and Phagocytosis to Tumor Cells

#### CD47

Macrophages never assault ordinary cells, since CD47 interacts with SIRP-1, a protein on its surface, and sends a negative signal back to macrophages, avoiding them from phagocytizing the target cells (Okazawa et al., 2005). Anti-CD47 antibody can block the interaction between CD47 and SIRPα, allowing phagocytes to selectively engulf tumor cells without damaging the surrounding healthy cells in melanoma and pediatric cancer (Uluçkan et al., 2009; Ridler, 2017; Hayat et al., 2020). *In vitro*, CD47 blockade triggered phagocytosis of cancer cells by macrophages, which has been thoroughly proved using microscopy and flow cytometry. *In vivo*, treatment of mice with liposomal clodronate, which exhausted macrophages, abolished the tumoricidal effects of CD47-blocking treatments, demonstrating macrophages were essential for robust anti-tumor responses (Xia et al., 2020). One inquiry supported that using a CpG oligodeoxynucleotide, a Toll-like receptor 9 agonist, to trigger macrophages could modify the metabolism of central carbon and strengthen the engulfment of CD47^+^ cancerous cells in pancreatic ductal adenocarcinoma models (PDAC) (Liu M. et al., 2019). Furthermore, *in situ* phagocytosis has been seen in leukemia, colon cancer, and breast cancer models (Weiskopf, 2017). However, a series of biosafety problems such as anemia should not be ignored.

### Regulating the Polarization of TAMs

To reverse the polarization of TAMs, targeting the cytokines in TME is another potential method. The Th1 cytokine, IFN-γ could fuel macrophages to polarize toward M1 phenotype. On the other hand, M2 polarization was first discovered as a reaction to Th2 cytokine IL-4 (Stein et al., 1992). Anti-inflammatory molecules including glucocorticoids, IL-4, IL-13, and IL-10 were proved to be direct inhibitors of classical macrophage activation, since they caused distinct M2 activation programs (Biswas and Mantovani, 2010). Ramesh et al. (2019) reported that M2 macrophages were repolarized to M1 phenotype and demonstrated inferior phagocytic capabilities after self-assembled dual-inhibitor-loaded nanoparticles (DNTs). This kind of DNTs was designed to “target” M2 macrophages while concurrently inhibiting CSF1R and SHP2 pathways in breast cancer and melanoma mouse models. The inhibition of Class IIa histone deacetylase (HDAC) in breast tumors reduced monocyte responses to CSF1 and CSF2 *in vitro* and caused intensely phagocytic and stimulatory macrophages to be recruited and differentiated (Guerriero et al., 2017; Guerriero, 2018).

### Activating the Ablation of TAMs

#### Legumain

Legumain, an asparaginyl endopeptidase, significantly expressed on M2 macrophages responding to the stimulation of Th2 cytokines in the TME. Legumain-ablated TAMs that directly triggered by a doxorubicin-based pro-drug could greatly inhibited tumor progress and metastases in breast carcinoma mouse models (Lin et al., 2013). Besides, alanine-alanine-asparagine (AAN), a substrate of endoprotease legumain, has also been identified as a specific ligand for TAMs (Liu Z. et al., 2014). To integrate AAN-based TAM-targeting and iRGD-based vascular and tumor permeability activities, nRGD was created by appending the substrate of endoprotease legumain (AAN) to the tumor homing peptide (iRGD). After loaded with Doxorubicin, liposomes that revised with nRGD (nRGD-Lipo-Dox) gained new skills by regulating TME with TAMs depletion, which explained the improved anti-tumor potency and long-term antiangiogenic effect in 4T1 breast cancer mice (Song et al., 2016).

#### Mannose Receptor

Macrophage mannose receptor, also called cluster of differentiation 206 (CD206), is a key promoter of tumor progression. Depleting CD206^+^ TAMs is a critical approach to cancer therapy (De Vlaeminck et al., 2019). TPE-Man, a theranostic probe with mannose moieties attached to a red-emissive AIE (aggregation-induced emission)-active photosensitizer, pinpointed the over-expressed mannose receptor on TAMs and effectively eradicated TAMs when exposed to white light irradiation, similar to the mannose-receptor antibody (Gao et al., 2019).

### Engineering CAR Macrophages With Tumor Specificity

Recently, adapting CAR strategies to macrophages have been demonstrated profitable. CAR macrophages (CAR-Ms) were designed to release pro-inflammatory cytokines and chemokines, convert M2 macrophages to M1, deliver a pro-inflammatory TME and increase T cells’ anti-tumor activity (Morrissey and Williamson, 2018; Klichinsky and Ruella, 2020).

## Conclusion

Over the last few years, with the constant emergence of modern technologies such as CRISPR/Cas9-mediated target gene activation (Xie et al., 2019; Morimoto and Nakazawa, 2021) and single cell RNA sequencing (Tirosh and Suvà, 2018), the characterization of GBM genome, epigenome, transcriptome revealed multiple subtypes and high inter- and intra-tumor heterogeneity of GBM (DeCordova et al., 2020). Glioma is a highly complex systemic disease that develops as a product of long-term Tumor/TME crosstalk between glioma cells and their local and distant microenvironments. TAMs, as one of the largest number of inflammatory cells in TME, perform a more fundamental and sophisticated character in glioma progression than previously thought (Li et al., 2021). Despite recent advances in T cell-based glioma therapy, accumulating evidence has showed that immunosuppressive microenvironment represents the main obstacle to maximize the effect of immunotherapies (Kwok and Okada, 2020; Mathewson et al., 2021). In view of the multi-dimensional influence of TAMs on tumor immunotherapies, TAMs, as the dominant immune cells in TME, are still indispensable targets for glioma therapy. So far, people have found that TAMs participate in the development and progression of glioma, and regulate tumor growth, invasion, angiogenesis, origin of GSCs and immune response. For these functions, numerous of experimental TAM-targeting strategies, such as macrophage elimination, recruitment inhibition and reprogramming, have been gradually developed. Some progress has been made in clinical and preclinical studies, but the prognosis of glioma patient is still unsatisfactory. Therefore, the function of TAMs in tumor progression needs more overall comprehension as well as the TAM-targeting strategies still need further development (Cassetta and Pollard, 2018). At present, there are still some questions that need to be forwarded.

(1) Recent data acquired utilizing unbiased large-scale techniques have discriminated that TAMs are not a simple binary M1-M2 typing, the intertwined relationships among each macrophage sub-populations’ location, subphenotype, polarization, dynamic changes and heterogeneity, glioma’s development and the efficacy of immunotherapy need to be fully unraveled by experimental research and clinical trials (Guillot and Tacke, 2019).

(2) Find highly specific molecular on TAMs that may be used as anti-glioma therapeutic objectives, to potentially low toxicity and side effects for successful therapy.
(3) The interaction between macrophages and other stromal cells should be further clarified to better understand the inter- and intra-driving force of the generation and maintenance of local glioma immunosuppressive microenvironment.
(4) What is the significance of macrophage subsets expressing different checkpoint receptors, such as PD-1 (Saha et al., 2017), T Cell Immunoglobulin and ITIM domain (TIGIT) (Chauvin and Zarour, 2020), or Lymphocyte-activation-gene-3 (LAG-3) (Maruhashi and Sugiura, 2020)? Is it possible to employ them to support T cell-targeting therapy?
(5) Governing the equilibrium between anti-cancer inflammatory response and pro-cancer inflammatory response is still an inherent problem to be solved.
(6) Recent multiple subclusters of TAMs have been established by single-cell RNA sequencing, suggesting that macrophage functionality is in a continuum of states. How to make TAM-targeting anti-glioma drugs get delivered through the BBB to act at the right point at the right time, and eliminate “bad” tumor-promoting TAMs without killing “good” macrophage in surrounding normal tissues?

In conclusion, at the genetic and immunological levels, the complicated and dramatic heterogeneity of malignant gliomas remains a profound challenge. As the dominant immune cells and indispensable target in immunologically cold tumor–glioma, TAMs’ characteristics and their interaction with TME, combined with the progress of diagnosis and treatment technology, need more comprehensively understanding. It is believed that targeting TAMs residing in TME in various ways for anti-glioma treatment strategy, in combination with the introduction of other therapeutic approaches, such as the recently discovered “pan cancer therapy,” combining SIRPα macrophage-based therapy with radiotherapy, miraculously activated tumor antigen-specific cytotoxic T cell in a broad spectrum of tumors including those at late-stage with low immunogenicity and metastases (Bian and Shi, 2021), may offer more and more innovative efficacious therapies for future glioma treatment and can be used as a realistic remedy for cancer patients.

## Author Contributions

All authors listed have made a substantial, direct and intellectual contribution to the work, and approved it for publication.

## Conflict of Interest

The authors declare that the research was conducted in the absence of any commercial or financial relationships that could be construed as a potential conflict of interest.
